# Methods to Calculate the Heat Index as an Exposure Metric in Environmental Health Research

**DOI:** 10.1289/ehp.1206273

**Published:** 2013-08-09

**Authors:** G. Brooke Anderson, Michelle L. Bell, Roger D. Peng

**Affiliations:** 1 Department of Biostatistics, Johns Hopkins Bloomberg School of Public Health, Baltimore, Maryland, USA; 2 School of Forestry and Environmental Studies, Yale University, New Haven, Connecticut, USA

## Abstract

Background: Environmental health research employs a variety of metrics to measure heat exposure, both to directly study the health effects of outdoor temperature and to control for temperature in studies of other environmental exposures, including air pollution. To measure heat exposure, environmental health studies often use heat index, which incorporates both air temperature and moisture. However, the method of calculating heat index varies across environmental studies, which could mean that studies using different algorithms to calculate heat index may not be comparable.

Objective and Methods: We investigated 21 separate heat index algorithms found in the literature to determine *a*) whether different algorithms generate heat index values that are consistent with the theoretical concepts of apparent temperature and *b*) whether different algorithms generate similar heat index values.

Results: Although environmental studies differ in how they calculate heat index values, most studies’ heat index algorithms generate values consistent with apparent temperature. Additionally, most different algorithms generate closely correlated heat index values. However, a few algorithms are potentially problematic, especially in certain weather conditions (e.g., very low relative humidity, cold weather). To aid environmental health researchers, we have created open-source software in R to calculate the heat index using the U.S. National Weather Service’s algorithm.

Conclusion: We identified 21 separate heat index algorithms used in environmental research. Our analysis demonstrated that methods to calculate heat index are inconsistent across studies. Careful choice of a heat index algorithm can help ensure reproducible and consistent environmental health research.

Citation: Anderson GB, Bell ML, Peng RD. 2013. Methods to calculate the heat index as an exposure metric in environmental health research. Environ Health Perspect 121:1111–1119; http://dx.doi.org/10.1289/ehp.1206273

## Introduction

Research that addresses health effects of weather-related heat exposure is critical both to limit present-day dangers from heat and also to prepare for future weather. Heat waves can produce catastrophic death tolls, including > 14,000 excess deaths during the 2003 French heat wave (Hémon et al. 2003), as well as increased risk of hospitalizations and adverse birth outcomes (e.g., Anderson et al. 2013; Basu et al. 2010). Under climate change, heat waves are expected to be more frequent and severe (Meehl and Tebaldi 2004). Beyond heat–health research, numerous other environmental health studies assess exposure to outdoor heat as a potential confounder (e.g., research on air pollution and health).

To estimate heat exposure, many environmental health studies use indices meant to capture the combined experience of several weather factors, such as the Universal Thermal Climate Index (UTCI 2012) and the humidex, which is used by Canada’s weather office (Environment Canada 2013).One of the most popular indices for environmental health research is Steadman’s apparent temperature (Steadman 1979a, 1979b, 1984), a version of which provides the basis for heat advisories in many U.S. communities [National Oceanic and Atmospheric Administration (NOAA) 2009]. Steadman’s apparent temperature translates current weather conditions (air temperature and air moisture in the most basic formulations) into the air temperature that would “feel” the same to humans if dew point temperature were 14.0°C/57.2°F (Rothfusz 1990; Steadman 1979a). By expressing weather conditions in terms of the equivalent temperature if dew point temperature were 14°C, Steadman translated combinations of air moisture and temperature [and other factors such as wind speed and sun radiation, in his original papers (Steadman 1979a, 1979b)] into a single scale, measured in the same units as air temperature. This index, particularly the simplified version that relies only on air temperature and moisture (Steadman 1979a), is often also called the “heat index” [here, we use “apparent temperature” to describe values originally presented in the tables by Steadman (1979a), whereas we use “heat index” to describe values generated by algorithms approximating Steadman’s original apparent temperature values (Ahrens 2007)].

Apparent temperature was developed to measure thermal comfort rather than to study human health (Steadman 1994). However, it has become a popular exposure metric in environmental health, particularly in its approximated “heat index” form. The U.S. National Weather Service (NWS) has linked different heat index values to environmental health threats [e.g., a heat index of 40.6°C/105°F indicates “danger” of heat-related disorders (NOAA 2012)], and the NWS uses heat index for its excessive heat warnings (NOAA 2009). Additionally, the heat index is widely used in environmental health research, including studies of air pollution exposures (e.g., Zanobetti and Schwartz 2005), outdoor temperature exposures (e.g., Barnett et al. 2010; Fletcher et al. 2012), and development of synoptic-scale heat warning systems (Sheridan and Kalkstein 2004; Smoyer-Tomic and Rainham 2001). The heat index has been used as a measure of heat exposure in studies throughout the world, including in studies of the United States (e.g., Zanobetti and Schwartz 2006), cities throughout Europe (e.g., Michelozzi et al. 2009), Australia (Khalaj et al. 2010), Bangladesh (Burkart et al. 2011), South Korea (Kysely and Kim 2009), and several Central and South American cities (Bell et al. 2008).

**Figure 1:**
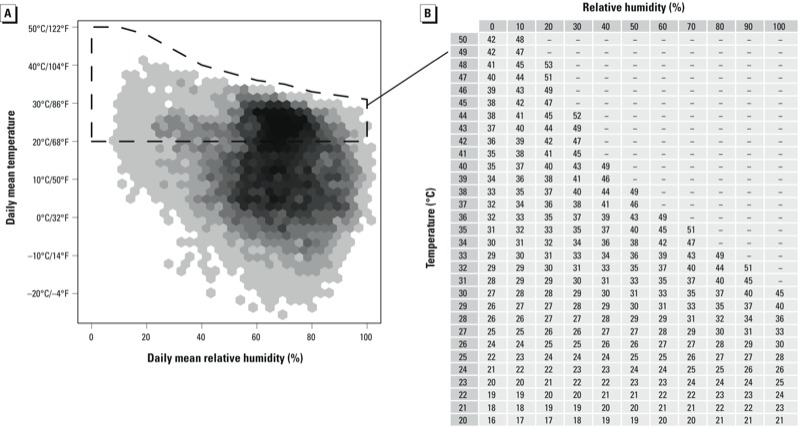
Distributions of daily temperature and relative humidity in U.S. state capitals in 2011 (*A*) and data from Steadman’s original apparent temperature table (*B*) (Steadman 1979a), which has been reformatted to correspond with the weather distribution graph and gives apparent temperature values in degrees Celsius. For the distribution graph (*A*), darker areas indicate more days with the given weather, and white indicates no days with those weather conditions in the U.S. state capitals in 2011. Weather conditions covered by Steadman’s table for air temperature and relative humidity are indicated by the dotted line. Data from Steadman (1979a), ^©^American Meteorological Society, are used with permission.

**Figure 2:**
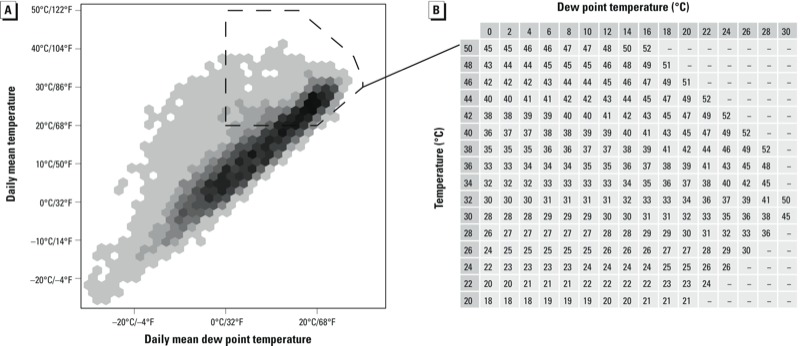
Distributions of daily temperature and dew point temperatures in U.S. state capitals in 2011 (*A*) and data from Steadman’s original apparent temperature table (*B*) (Steadman 1979a), which has been reformatted to correspond with the weather distribution graph and gives apparent temperature values in degrees Celsius. For the distribution graph (*A*), darker areas indicate more days with the given weather, and white indicates no days with those weather conditions in the U.S. state capitals in 2011. Weather conditions covered by Steadman’s table for air temperature and dew point temperature are indicated by the dotted line. Data from Steadman (1979a), ^©^American Meteorological Society, are used with permission.

Calculating apparent temperature using Steadman’s original equations requires iterating multiple equations that describe heat and moisture transfer until a final equation converges (Steadman 1979a). Steadman performed this calculation for specific combinations of air temperature and moisture (relative humidity or dew point temperature). He published these values in two tables (Steadman 1979a; reproduced with permission in Figures 1B and 2B), which can be used to look up apparent temperature for specific combinations of air temperature and moisture. Within each table in Figures 1 and 2, each row represents a specific temperature, and moving across each row shows how heat index changes at a constant temperature with increasing air moisture. Extensive details are given in the original paper that developed the heat index (Steadman 1979a) to describe how physiological heat-regulation principles were used to incorporate both air temperature and moisture to determine heat index values for specific weather conditions.

Although both tables give heat index values based on air temperature and moisture, the two tables are based on two different measures of air moisture—relative humidity and dew point temperature—and the two tables cover different ranges of possible weather. Together, the tables cover most hot weather experienced in the United States; as an illustration, Figures 1A and 2A show the joint distribution of daily mean air temperature and air moisture for the 50 U.S. state capitals in 2011, and weather conditions covered by each of Steadman’s two original tables (1979a) are highlighted. Conversely, these tables do not cover cool and cold weather (Figures 1, 2).

As alternatives to looking up heat index values from Steadman’s tables, heat index algorithms are numerically derived equations that attempt to reproduce the values in these tables. These algorithms are attractive alternatives to Steadman’s tables for environmental health research, because they can *a*) efficiently calculate a long series of heat index values based on observations of air temperature and moisture, *b*) interpolate for weather conditions between the cells of the original tables, *c*) be applied to all weather conditions, and *d*) unify extreme temperature for singular heat events (such as heat waves) across many jurisdictions.

**Figure 3:**
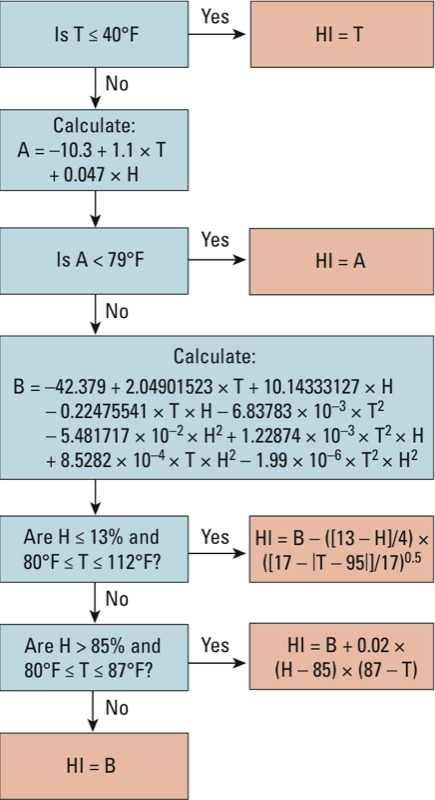
Algorithm used by the NWS online heat index (HI) calculator (NWS 2011) to determine heat index based on air temperature in degrees Fahrenheit (T) and relative humidity in percent (H).

Although such algorithms are commonly used to calculate heat index values for environmental research, the specific heat algorithm used varies across studies. In a search of environmental literature, we identified 21 different heat index algorithms (Table 1), including simple equations with single terms for air temperature and moisture (algorithms 4, 13–14, 19, and 21; Table 1), equations with air temperature and moisture (i.e., dew point temperature, relative humidity, water vapor pressure) as exponential terms (algorithms 2 and 3), multiterm equations with air temperature and moisture included up to quadratic terms (algorithms 16 and 17), and algorithms with correction factors for certain weather conditions (algorithms 5–12 and 15). In environmental health research, simpler heat index algorithms are typical (e.g., Barnett et al. 2010; Halonen et al. 2011a; Smoyer-Tomic and Rainham 2001; Vaneckova et al. 2011; Zanobetti and Schwartz 2005). More complex algorithms are more common in climatology studies (e.g., Fischer and Schär 2010; Oka 2011), although some environmental health studies have used these more complex algorithms as well (e.g., Fletcher et al. 2012; Lajinian et al. 1997; Tam et al. 2008). The NWS uses its own complex algorithm for forecasts and heat warnings (Figure 3) and has created a website that calculates heat index using this algorithm, although only for one heat index value at a time (NWS 2011).

**Table 1:** Heat index algorithms that have been used in environmental research.

No.	Algorithm	Reference
1	NWS algorithm (Figure 3)	NWS 2011^*a*^
2	*HI*_*C*_ = *T*_*C*_ – 1.0799*e*^0.03755^^*T*^^*C*^(1 – *e*^0.0801(^^*D*^^*C*^^* – *^^14)^)	Schoen 2005^*a*^
3	*HI*_*F*_ = *T*_*F*_ – 0.9971*e*^0.02086^^*T*^^*F*^(1–*e*^0.0445(^^*D*^^*F*^^* – *^^57.2)^)	Schoen 2005^*a*^
4	*HI*_*C*_ = –1.3 + 0.92*T*_*C*_ + 2.2*e*_*S*_	Gaffen and Ross 1999; Steadman 1984^*a*^
5	*HI*_*F*_ = –42.379 + 2.04901523*T*_*F*_ + 10.14333127*H *– 0.22475541*T*_*F*_*H *– (6.83783 × 10^–3^)*T*_*F*_^2^ – (5.481717 × 10^–2^)*H*^2^ + (1.22874 × 10^–3^)*T*_*F*_^2^*H* + (8.5282 × 10^–4^)*T*_*F*_*H*^2^ – (1.99 × 10^–6^)*T*_*F*_^2^*H*^2^. Correction factor: *HI*_*F*_ = *T*_*F*_ when *T*_*F*_ ≤ 80°F or *H* ≤ 40%	El Morjani et al. 2007^*a*^; Oka 2011
6	*HI*_*F*_ = –42.379 + 2.04901523*T*_*F*_ + 10.14333127*H *– 0.22475541*T*_*F*_*H *– (6.83783 × 10^–3^)*T*_*F*_^2^ – (5.481717 × 10^–2^)*H*^2^ + (1.22874 × 10^–3^)*T*_*F*_^2^*H* + (8.5282 × 10^–4^)*T*_*F*_*H*^2^ – (1.99 × 10^–6^)*T*_*F*_^2^*H*^2^. Correction factor: *HI*_*F*_ = *T*_*F*_ when *T*_*F*_ < 80°F or *H* < 40%	Fandoeva et al. 2009^*a*^
7	*HI*_*F*_ = –42.379 + 2.04901523*T*_*F*_ + 10.14333127*H *– 0.22475541*T*_*F*_*H *– (6.83783 × 10^–3^)*T*_*F*_^2^ – (5.481717 × 10^–2^)*H*^2^ + (1.22874 × 10^–3^)*T*_*F*_^2^*H* + (8.5282 × 10^–4^)*T*_*F*_*H*^2^ – (1.99 × 10^–6^)*T*_*F*_^2^*H*^2^. Correction factor: *HI*_*F*_ = *T*_*F*_ when *T*_*F*_ ≤ 78.8°F or *H* ≤ 39%	Di Cristo et al. 2007^*a*^; Rajib et al. 2011
8	*HI*_*F*_ = –42.4 + 2.049*T*_*F*_ + 10.14*H *– 0.2248*T*_*F*_*H *– (6.838 × 10^–3^)*T*_*F*_^2^ – (5.482 × 10^–2^)*H*^2^ + (1.229 × 10^–3^)*T*_*F*_^2^*H* + (8.528 × 10^–4^)*T*_*F*_*H*^2^ – (1.99 × 10^–6^)*T*_*F*_^2^*H*^2^. Correction factor: *HI*_*F*_ = *T*_*F*_ when *T*_*F*_ < 79°F	Johnson and Long 2004^*a*^
9	*HI*_*F*_ = 16.923 + 0.185212*T*_*F*_ + 5.37941*H *– 0.100254*T*_*F*_*H* + (9.4169 × 10^–3^)*T*_*F*_^2^ + (7.28898 × 10^–3^)*H*^2^ + (3.45372 × 10^–4^)*T*_*F*_^2^*H*– (8.14971 × 10^–4^)*T*_*F*_*H*^2^ + (1.02102 × 10^–5^)*T*_*F*_^2^*H*^2^ – (3.8646 × 10^–5^)*T*_*F*_^3^ + (2.91583 × 10^–5^)*H*^3^ + (1.42721 × 10^–6^)*T*_*F*_^3^*H* + (1.97483 × 10^–7^)*T*_*F*_*H*^3^ – (2.18429 × 10^–8^)*T*_*F*_^3^*H*^2^ + (8.43296 × 10^–10^)*T*_*F*_^2^*H*^3^ – (4.81975 × 10^–11^)*T*_*F*_^3^*H*^3^ + 0.5. Correction factor: *HI*_*F*_ = *T*_*F*_ when *T*_*F*_ < 75°F	Robinson 2001^*a*^
10	*HI*_*C*_ = –8.784695 + 1.61139411*T*_*C*_ + 2.338549*H *– 0.14611605*T*_*C*_*H *– (1.2308094 × 10^–2^)*T*_*C*_^2^ – (1.6424828 × 10^–2^)*H*^2^ + (2.211732 × 10^–3^)*T*_*C*_^2^*H* + (7.2546 × 10^–4^)*T*_*C*_*H*^2^ – (3.582 × 10^–6^)*T*_*C*_^2^*H*^2^. Correction factor: *HI*_*C*_ = *T*_*C*_ when *T*_*C*_ ≤ 20°C	Blazejczyk et al. 2012^*a*^
11	*HI*_*F*_ = –42.4 + 2.05*T*_*F*_ + 10.1*H *– 0.255*T*_*F*_*H *– (6.84 × 10^–3^)*T*_*F*_^2^ – (5.48 × 10^–2^)*H*^2^ + (1.23 × 10^–3^)*T*_*F*_^2^*H* + (8.53 × 10^–4^)*T*_*F*_*H*^2^ – (1.99 × 10^–6^)*T*_*F*_^2^*H*^2^. Correction factor: *HI*_*F*_ = *T*_*F*_ when *T*_*F*_ ≤ 80°F or *H* ≤ 40%	Patricola and Cook 2010^*a*^
12	*HI*_*C*_ = –2.719 + 0.994*T*_*C*_ + 0.016*D*_*C*_^2^. Correction factor: *HI*_*C*_ = *T*_*C*_ when *T*_*C*_ < 25°C	Smoyer-Tomic and Rainham 2001^*a*^
13	*HI*_*C*_ = –2.653 + 0.994*T*_*C*_ + 0.0153*D*_*C*_^2^	Analitis et al. 2008; Basara et al. 2010; Halonen et al. 2011a, 2011b; Kuchcik 2006; Mbanu et al. 2007; Michelozzi et al. 2007, 2009; O’Neill et al. 2003; Rich et al. 2008; Schneider et al. 2008; Zanobetti and Schwartz 2005^*a*^, 2006
14	*HI*_*C*_ = –2.719 + 0.994*T*_*C*_ + 0.016*D*_*C*_^2^	Perry et al. 2011^*a*^
15	*HI*_*F*_ = –42.379 + 2.049015*T*_*F*_ + 10.1433*H *– 0.2248*T*_*F*_*H *– (6.83783 × 10^–3^)*T*_*F*_^2 ^– (5.4817 × 10^–2^)*H*^2^ + (1.229 × 10^–3^)*T*_*F*_^2^*H* + (8.528 × 10^–4^)*T*_*F*_*H*^2^ – (1.99 × 10^–6^)*T*_*F*_^2^*H*^2^. Correction factor: *HI*_*F*_ = *T*_*F*_ when *T*_*F*_ < 57°F	Tam et al. 2008^*a*^
16	*HI*_*F*_ = –42.379 + 2.04901523*T*_*F*_ + 10.14333127*H *– 0.22475541*T*_*F*_*H *– (6.83783 × 10^–3^)*T*_*F*_^2^ – (5.481717 × 10^–2^)*H*^2^ + (1.22874 × 10^–3^)*T*_*F*_^2^*H* + (8.5282 × 10^–4^)*T*_*F*_*H*^2^ – (1.99 × 10^–6^)*T*_*F*_^2^*H*^2^	Rothfusz 1990^*a*^
17	*HI*_*C*_ = –8.7847 + 1.6114*T*_*C*_**– 0.012308*T*_*C*_^2^ + *H*[2.3385 – 0.14612*T*_*C*_ + (2.2117 × 10^–3^)*T*_*C*_^2^] + *H*^2^[–0.016425 + (7.2546 × 10^–4^)*T*_*C*_ + (–3.582 × 10^–6^)*T*_*C*_^2^]	Fischer and Schär 2010^*a*^
18	*HI*_*C*_ = *T*_*C*_**– 0.55 × (1 – 0.001*H*)(*T*_*C*_**– 14.5)	Costanzo et al. 2006^*a*^
19	*HI*_*C*_ = 2.719 + 0.994*T*_*C*_ + 0.016*D*_*C*_^2^	Smoyer 1998a^*a*^, 1998b
20	*HI*_*F*_ = *T*_*F*_**– {[0.55 – 0.55(*H*/100)]*T*_*F*_**– 58}	Lajinian et al. 1997^*a*^
21	*HI*_*C*_ = –2.653 + 0.994*T*_*C*_ + 0.368*D*_*C*_^2^	Basara et al. 2010^*a*^; Vaneckova et al. 2011
Abbreviations: *D*_C_, dew point temperature in degrees Celsius; *D*_F_, dew point temperature in degrees Fahrenheit; *e*_S_, water vapor pressure in kilopascals; *H*, humidity in percent; *HI*_C_, heat index in degrees Celsius; *HI*_F_, heat index in degrees Fahrenheit; *T*_C_, air temperature in degrees Celsius; *T*_F_, air temperature in degrees Fahrenheit. ^***a***^Earliest publication of the algorithm found through our research; in some but not all cases, this is the original source of the algorithm.		

Given the variety of heat index algorithms in environmental research, it is unclear whether *a*) all heat index algorithms produce heat index values that are consistent with the theoretical concepts underlying Steadman’s apparent temperature, and *b*) whether different algorithms generate similar heat index values. If different algorithms generate dissimilar heat index values, it may be problematic to compare results (e.g., meta-analysis) for studies that use different heat index algorithms or translate results to heat index values reported in meteorological forecasts.

## Methods

*Data*. To investigate the performance of different heat index algorithms under realistic U.S. weather conditions, we collected daily 2011 weather data, including mean air temperature, mean dew point temperature, and mean relative humidity, for the 50 U.S. state capitals from Weather Underground’s historical weather data (Weather Underground 2012). This historical data come from weather measurements from automated airport weather stations (airport identification numbers for each state capital given in Supplemental Material, Table S1). For quality control, we checked data in all cities for any unreasonable temperature values [temperatures > 50°C or < –40°C (Kloog et al. 2012)] and found no problematic observations.

*Overview of analysis*. We investigated 21 separate heat index algorithms found in environmental studies (Table 1), using Google Scholar (http://scholar.google.com) keyword searches for “heat index” and “apparent temperature.” Our only exclusion criteria in selecting algorithms were that the algorithm equation be explicitly stated in the paper and that the algorithm required inputs only of air temperature and air moisture.

*Agreement with Steadman’s apparent temperature*. We first analyzed whether each algorithm produced heat index values consistent with Steadman’s original apparent temperature. All discussion of Steadman’s original tables in this section refer to Steadman (1979a). Our intent with this analysis was not to identify a “best” algorithm for approximating heat index, but rather to determine whether algorithms used in the literature produce values that reasonably agree with the theoretical concepts underlying Steadman’s original apparent temperature. We used two criteria:

Within the weather range of Steadman’s original tables, the algorithm gives values similar to Steadman’s original apparent temperature values; and
Outside the weather range of Steadman’s original tables, the algorithm gives values that reasonably agree with the theoretical concepts underlying Steadman’s original temperature calculations.
For each of these criteria, we developed reasonable metrics to test the criterion for the 21 separate heat index algorithms.

Agreement for weather conditions within Steadman’s original tables. Between Steadman’s two original tables (1979a), heat index values calculated using Steadman’s original physiological models are available for air temperature between 20°C/68°F and 50°C/122°F, relative humidity between 0% and 100%, and dew point temperature between 0°C/32°F and 30°C/86°F (Figures 1B, 2B). For each of the 21 algorithms, we used two different methods to quantify how well heat index values generated by the algorithm agree with values in these tables for these weather conditions.

*Unweighted metric.* We first investigated each cell in Steadman’s original tables and calculated the absolute difference between Steadman’s value and the heat index value calculated by the algorithm, and then averaged these absolute differences across all table cells (“unweighted metric”) for both of Steadman’s two original tables (Figures 1B, 2B) using the following process:

For each of Steadman’s two tables (Figures 1B, 2B), we used the algorithm to calculate heat index for each table cell’s weather conditions (e.g., for the relative humidity table: air temperature of 20°C and relative humidity of 0%; air temperature of 20°C and relative humidity of 10%).
For each table cell, we calculated the difference between the heat index value calculated by the algorithm and the apparent temperature value in the original table for those weather conditions.
We averaged these table cell-specific absolute differences.

This metric (in degrees Celsius) measures the average difference between the heat index values calculated by an algorithm and each of Steadman’s two original apparent temperature tables.

*Weighted metric.* As a second metric (weighted metric), we calculated these average absolute differences with each table cell weighted by how often the weather conditions represented by that cell occurred in the 50 U.S. state capitals in 2011. Steadman’s tables cover some weather conditions that are very rare in the United States (e.g., relative humidity < 10%; Figure 1). This weighted metric acknowledges that, for many applications, inconsistencies are less important for weather conditions that rarely or never occur than for conditions that occur frequently.

For this metric, we determined appropriate weights for each cell in Steadman’s tables based on the frequency of weather conditions in the 50 U.S. state capitals in 2011 using the following process:

For each daily weather observation in the 50 U.S. state capitals in 2011, we linked the weather to the appropriate cell in Steadman’s table. For example, for a weather observation with air temperature 25.4°C and relative humidity 43%, we matched the observation with the table cell for air temperature 25°C and relative humidity 40%. Weather observations outside of the range of the tables were excluded for this analysis.
We next determined weights for each table cell based on the frequency of the weather conditions described by each cell. We counted the number of weather observations that corresponded to each cell of the table and then divided these cell-specific counts by the total number of weather observations within the weather ranges of the table. This resulted in fractions to describe the comparative frequency of each table cell based on 2011 weather in the 50 U.S. state capitals (Figures 1A, 2A).

We then calculated this weighted metric using the same process used for the unweighted metric, but with the difference in each cell weighted by the weather frequency weights for the table.

Agreement for weather conditions outside Steadman’s tables. As a second criterion, we tested whether a heat index algorithm cohered with the concepts behind Steadman’s apparent temperature at weather conditions beyond those given in Steadman’s original tables. In the 50 U.S. state capitals, there were no days in 2011 with weather warmer than values given in Steadman’s tables (Figures 1A, 2A). However, many weather observations were cooler or less humid than the conditions given in Steadman’s table. Because many environmental health studies of temperature and health use year-round data (e.g., Anderson and Bell 2009), it is important to determine whether heat index algorithms perform in unanticipated ways when applied to data sets that include cooler weather.

At cooler air temperatures, sweat evaporation is not an important avenue of heat transfer from the human body (Wenger 2003), so apparent temperature should change little with air moisture at these lower temperatures (Steadman 1984). Therefore, during cool or cold weather, heat index values should equal or be very similar to air temperature.

As a test, we measured the average absolute difference between the heat index values calculated by each algorithm and air temperature for cool and cold days (air temperature < 20°C/68°F, the cutoff for Steadman’s original tables) in the U.S. state capitals in 2011. For this metric, we first created a subset of cool and cold weather in the United States. For each of these observations, we calculated the absolute difference between air temperature and the value of heat index measured by the algorithm and then took the average value of these absolute differences. This metric, in degrees Celsius, represents the average absolute difference between values from a heat index algorithm and air temperature during cool and cold days.

*Agreement between different algorithms*. We next compared heat index values generated by different algorithms. If different algorithms produce dissimilar or poorly correlated heat index values, environmental health effect estimates may not be comparable across studies using different algorithms.

We measured the Pearson correlation coefficient between each pair-wise combination of the 21 heat index algorithms. To measure this correlation, we used each of the two heat index algorithms to calculate heat index values for all observations in our data set of daily weather in 2011 in the U.S. state capitals. We then measured the correlation between heat index values determined by the two algorithms.

*Heat index algorithm software*. Finally, using the NWS’s heat index algorithm, we developed open-source software to allow researchers to generate heat index values for large weather data sets within the R statistical platform (R Project for Statistical Computing, Vienna, Austria).

## Results

*Agreement with Steadman’s apparent temperature*. Agreement for weather conditions within Steadman’s original tables. Many of the algorithms produced heat index values very similar to the values in Steadman’s original tables within the relevant weather conditions, as judged by both the unweighted and weighted metrics (Table 2). For example, the NWS algorithm (Figure 3), which performed best on these metrics, provided heat index values that were, on average, within 0.4°C/0.7°F of the original Steadman values for both relative humidity and dew point temperature tables (algorithm 1; Table 2).

**Table 2:** Metrics (°C) describing how well different heat index algorithms cohere with the original concepts of Steadman’s apparent temperature.

Algorithm^*a*^	Unweighted metric, compared with Steadman’s relative humidity table^*b*^	Weighted metric, compared with Steadman’s relative humidity table^*c*^	Unweighted metric, compared with Steadman’s dew point temperature table	Weighted metric, compared with Steadman’s dew point temperature table	Compared with air temperature during mild or cold weather^*d*^
1	0.4	0.3	0.4	0.2	0.8
2	0.8	0.4	0.7	0.3	0.8
3	0.8	0.4	0.7	0.3	0.8
4	1.5	0.7	1.4	0.5	0.4
5	1.9	0.6	1.7	0.6	0.0
6	1.7	0.6	1.7	0.6	0.0
7	1.7	0.6	1.7	0.6	0.0
8	0.8	0.6	0.7	0.6	0.0
9	0.9	0.7	0.8	0.7	0.0
10	1.0	1.0	0.8	0.9	0.0
11	2.2	1.1	1.9	0.9	0.0
12	22.8	1.2	1.0	1.0	0.0
13	25.9	1.3	1.0	1.1	1.9
14	27.0	1.4	1.1	1.2	2.0
15	1.2	1.1	1.0	1.5	2.1
16	1.2	1.1	1.0	1.5	42.6
17	1.2	1.1	1.0	1.5	42.6
18	9.5	6.6	11.3	5.9	4.2
19	31.6	6.6	5.5	6.3	3.8
20	14.7	22.4	12.0	22.0	27.6
21	682.3	114.7	78.5	95.2	23.4
^***a***^Algorithm numbers correspond to algorithm numbers given in Table 1. ^***b***^Average absolute difference between apparent temperature values in cells of Steadman’s original tables and heat index values calculated using the given algorithm for the weather conditions described by each table cell. ^***c***^The weighted metrics give the same measurement of absolute differences, but with the average weighted by how frequently the weather conditions described by each table cell were experienced in the 50 U.S. state capitals in 2011. ^***d***^Average absolute difference between air temperature and heat index generated by the algorithm for all days in 2011 in the U.S. state capitals with air temperature < 20°C/68°F.					

A few algorithms (e.g., algorithms 12–14; Table 2) had large average differences with Steadman’s relative humidity table for the unweighted metric, but small differences with the dew point temperature table based on the unweighted metric and for both tables based on the weighted metrics. Other algorithms (algorithms 18–21; Table 2) differed substantially from original apparent temperature values as judged by both weighted and unweighted metrics for both tables, with an average difference from original table values of > 5°C/9°F for both metrics.

Agreement for weather conditions outside Steadman’s tables. Some heat index algorithms include correction factors for cool temperature (e.g., algorithms 5–12 and 15; Table 1). These universally produced heat index values similar or equal to air temperature during cool and cold weather (Table 2). Algorithms without correction factors differed in their performance at weather conditions outside those given in Steadman’s tables. For example, algorithms 1–4 all produced heat index values very similar to air temperature values at cool and cold conditions, whereas algorithms 16 and 17 produced heat index values that, on average, differed > 40°C/72°F from air temperature values on cool and cold days (Table 2).

*Agreement between different algorithms*. Most algorithms produced well-correlated heat index values for daily weather in the 50 U.S. state capitals in 2011 (Table 3). For 16 algorithms, heat index values were either perfectly or almost perfectly positively correlated with each other, and most other algorithms gave strongly correlated heat index values (i.e., *r*_P_ > 0.90), except for three algorithms. One algorithm (21) generated heat index values that were only moderately correlated with most other algorithms (average *r*_P_ = 0.63; range, –0.23, 0.81). Two other algorithms (16–17) generated heat index values that were negatively correlated with most other algorithms (average correlation with other algorithms, –0.65; Table 3).

**Table 3:** Correlations between the heat index values calculated by each of the 21 algorithms.

Algorithm no.^*a*^	1	2	3	4	5	6	7	8	9	10	11	12	13	14	15	16	17	18	19	20	21
1	1	1	1	1	1	1	1	1	1	1	1	1	0.99	0.99	0.95	–0.76	–0.76	0.99	0.99	0.95	0.73
2	—	1	1	1	1	1	1	1	1	1	1	1	1	0.99	0.96	–0.77	–0.77	1	0.99	0.96	0.73
3	—	—	1	1	1	1	1	1	1	1	1	1	1	0.99	0.96	–0.77	–0.77	1	0.99	0.96	0.73
4	—	—	—	1	1	1	1	1	1	1	1	1	0.99	0.99	0.96	–0.78	–0.78	1	0.99	0.96	0.72
5	—	—	—	—	1	1	1	1	1	1	1	1	0.99	0.99	0.96	–0.77	–0.77	1	0.99	0.94	0.70
6	—	—	—	—	—	1	1	1	1	1	1	1	0.99	0.99	0.96	–0.77	–0.77	1	0.99	0.94	0.70
7	—	—	—	—	—	—	1	1	1	1	1	1	0.99	0.99	0.96	–0.77	–0.77	1	0.99	0.94	0.70
8	—	—	—	—	—	—	—	1	1	1	1	1	0.99	0.99	0.96	–0.78	–0.78	1	0.99	0.95	0.70
9	—	—	—	—	—	—	—	—	1	1	1	1	0.99	0.99	0.96	–0.77	–0.77	1	0.99	0.95	0.72
10	—	—	—	—	—	—	—	—	—	1	1	1	0.99	0.99	0.96	–0.77	–0.77	1	0.99	0.94	0.70
11	—	—	—	—	—	—	—	—	—	—	1	1	0.99	0.99	0.96	–0.79	–0.79	1	0.99	0.94	0.69
12	—	—	—	—	—	—	—	—	—	—	—	1	0.99	0.99	0.95	–0.76	–0.76	0.99	0.99	0.95	0.73
13	—	—	—	—	—	—	—	—	—	—	—	—	1	1	0.94	–0.72	–0.72	0.99	1	0.95	0.79
14	—	—	—	—	—	—	—	—	—	—	—	—	—	1	0.94	–0.71	–0.71	0.99	1	0.95	0.79
15	—	—	—	—	—	—	—	—	—	—	—	—	—	—	1	–0.79	–0.79	0.96	0.94	0.90	0.62
16	—	—	—	—	—	—	—	—	—	—	—	—	—	—	—	1	1	–0.80	–0.71	–0.78	–0.23
17	—	—	—	—	—	—	—	—	—	—	—	—	—	—	—	—	1	–0.80	–0.71	–0.78	–0.23
18	—	—	—	—	—	—	—	—	—	—	—	—	—	—	—	—	—	1	0.99	0.94	0.68
19	—	—	—	—	—	—	—	—	—	—	—	—	—	—	—	—	—	—	1	0.95	0.79
20	—	—	—	—	—	—	—	—	—	—	—	—	—	—	—	—	—	—	—	1	0.74
21	—	—	—	—	—	—	—	—	—	—	—	—	—	—	—	—	—	—	—	—	1
For each pair of algorithms, both algorithms were used to generate heat index values for daily weather from the 50 U.S. state capitals in 2011. The Pearson correlation between daily values from the two algorithms was then calculated and is presented here (values were correlated in time per station and then averaged over all stations). ^***a***^Columns and rows are marked by algorithm number, corresponding to algorithm numbers in Tables 1 and 2.																					

*Heat index algorithm software*. The NWS algorithm (Figure 3) agreed best with Steadman’s apparent temperature by all metrics considered and for both of Steadman’s original tables. This algorithm has the added advantage of familiarity, because heat index values generated with it are commonly reported in U.S. weather reports. To provide environmental health researchers a convenient way to use this algorithm, we developed *weathermetrics*, an R package that allows fast and easy calculation of heat index for weather data sets using the NWS’s heat index algorithm (Anderson and Peng 2012).

## Discussion

*Agreement with Steadman’s apparent temperature*. Agreement for weather conditions within Steadman’s original tables. Many of the algorithms tested generated heat index values that were very similar to values from Steadman’s original tables for weather conditions covered by the tables. However, some algorithms disagreed with Steadman’s tables for certain weather conditions. The weighted algorithms measured for this analysis are relevant for studies in any locations with weather conditions similar to those of the 50 U.S. state capitals in 2011 (Figures 1A, 2A). The unweighted algorithms do not rely on observed weather data and so are relevant for any location.

A few algorithms cohered poorly at extremely low relative humidity (< 10%), but agreed well with Steadman’s tables for all other weather (e.g., algorithms 12–14). As a result, these algorithms had large values for the unweighted metric for the relative humidity table—the only table that covers very low relative humidity (Table 2). At low relative humidity, dew point temperature can have a large negative value (e.g., at air temperature 10°C/50°F and relative humidity 5%, dew point temperature is –27.8°C/–18.0°F). These algorithms include dew point temperature squared (Table 1), and so give much higher heat index values than Steadman’s table when relative humidity is very low because the large negative dew point temperature squares to a large positive value.

This isolated issue is unlikely to be practically problematic, because such low relative humidity is rare. Indeed, these algorithms perform well under the weighted metric, which weights average differences by U.S. weather distributions (Table 2). For example, once table cells were weighted by weather distributions, heat index values given by algorithm 12 (Table 2) differed < 1.2°C/2.2°F on average from the original tables.

A few of the algorithms (algorithms 18–21) differed substantially from original table values under all metrics considered, with an average difference from original table values of > 5°C/9°F for both metrics (Table 2). These algorithms may introduce substantial errors in exposure measurements in environmental health studies by generating estimated heat index values several degrees different from the metric meant to be measured.

Agreement for weather conditions outside Steadman’s tables. Some heat index algorithms explicitly handle cool and cold temperatures with a correction factor that sets heat index to air temperature below a cut-off temperature (e.g., algorithms 5–12 and 15; Table 1). These algorithms all produced heat index values equal to air temperature at cool and cold temperatures and so performed perfectly on this criterion (Table 2).

Other heat index algorithms lack correction factors (Table 1). Several of these algorithms nonetheless performed well on this criterion, generating heat index values on average within 1°C/1.8°F of air temperature for cool and cold days (e.g., algorithms 1–4; Table 2). Others, however, performed poorly in cool or cold temperatures. For example, two algorithms (algorithms 16 and 17) produced heat index values that, on average, differed > 40°C/72°F from air temperature values on cool and cold days (Table 2). Although these two algorithms agreed well with Steadman’s apparent temperature at warmer temperatures, they would produce unreasonable heat index values in year-round data sets that include cooler weather. At cool temperatures, several of the negative terms that include temperature in algorithms 16 and 17 (Table 1), especially the fourth term, are much closer to zero for cold temperatures than for hot temperatures. Although for hot weather, these terms appropriately offset positive terms in the algorithms to give reasonable heat index values, these algorithms generate inappropriately high heat index values when temperature is cold.

Algorithms 16 and 17 differ from algorithms 5–8 mainly in that algorithms 16 and 17 lack correction factors to set heat index equal to air temperature at cool temperatures. Although these algorithms would all give very similar heat index values for weather data limited to warmer temperatures, our analysis indicates problematic heat index values when algorithms 16 and 17 are applied to year-round data that include cool and cold days.

In this analysis we investigated the performance of heat algorithms during weather conditions that are not covered by Steadman’s original tables (e.g., Figures 1, 2) but that are common in year-round weather data for temperate locations. These results suggest that the performance of heat index algorithms may vary by season, given seasonal changes in weather conditions, and that algorithms that perform without major concerns when applied to warm weather (e.g., algorithms 16 and 17) may be problematic in data sets that include year-round weather observations.

*Agreement between different algorithms.* Most pairs of algorithms produced well-correlated heat index values for daily 2011 weather in the 50 U.S. state capitals (Table 3). Two algorithms (16 and 17), however, generated heat index values that were negatively correlated with most other algorithms (average correlation with other algorithms, –0.65; Table 3). These two algorithms are problematic at cool and cold temperatures (Table 2), where they give heat index values that are much higher than air temperature. Therefore, when used with year-round data sets that include cool or cold weather, these algorithms may give heat index values that are not well correlated with those generated by other algorithms.

*Heat index algorithm software*. Previously, the NWS algorithm was considered too complex for general use in environmental health research (Smoyer-Tomic and Rainham 2001). However, because open-source statistical software such as R is increasingly popular in environmental health research, complex algorithms can now be more easily implemented. With permission from the NWS, we converted JavaScript code from their online heat index calculator (NWS 2011) into an R function that can be applied to large weather data sets, which we have included in the *weathermetrics* R package (Anderson and Peng 2012). We made this package freely available through the Comprehensive R Archive Network, with details and examples included in a vignette available with the package (http://cran.r-project.org/web/packages/weathermetrics/index.html).

**Figure 4:**
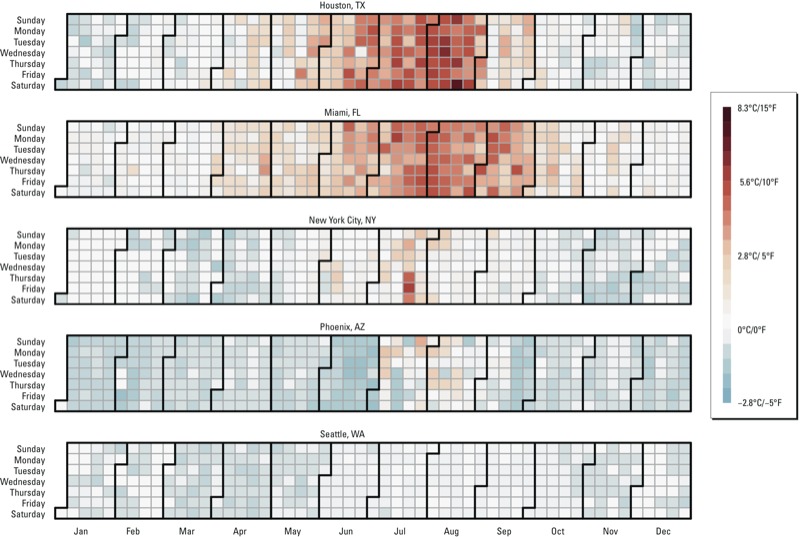
Daily differences between heat index and air temperature for each day in 2011 for five U.S. cities. Color shows heat index minus temperature for that day in 2011 in the specified city. Lighter colors indicate that heat index and air temperature were very similar. Darker red (blue) indicate heat index was higher (lower) than air temperature. The figure shows the difference in temperatures, not absolute temperatures.

*Additional considerations*. The heat index is frequently used to measure environmental heat exposure in environmental health studies, which prompted this study’s examination of heat index algorithms. However, in planning new research, a variety of other metrics (e.g., mean, maximum, or minimum temperature) can be used to measure heat exposure, and several additional considerations are important for deciding whether to use heat index rather than another exposure metric for environmental health research. First, although the heat index has conceptual appeal for environmental health research, in many communities temperature and heat index values rarely differ. For example, in dry cities such as Phoenix, Arizona, and cities with mild summers such as Seattle, Washington, the heat index and air temperature are almost identical throughout the year (Figure 4). In these cities, little is gained by using heat index rather than air temperature to measure exposure, other than comparability with other studies, which is still hindered by the variation in heat index formulations.

In other locations, heat index and air temperature differ more during summer (e.g., Houston, TX, and Miami, FL; Figure 4). However, throughout the United States, daily values of the two metrics are very strongly correlated (median city-specific correlation for the 50 U.S. state capitals in 2011, 0.996; range, 0.983–0.999). Given this close correlation, it is unlikely that results from studies using the two metrics will vary much. Indeed, several studies have tested the sensitivity of heat–health effect estimates to measuring exposure with heat index versus air temperature, and none found large differences in estimates (Anderson and Bell 2009; Barnett et al. 2010; Medina-Ramón et al. 2006; Vaneckova et al. 2011).

Regardless, most people have experienced how humidity can modify the “feel” of heat. Even though heat index and temperature are strongly correlated day to day in the cities considered here, and so time-series studies using the two metrics are likely to have similar quantitative results, some researchers may still have reasons to include in their studies time-series results based on heat index as well as temperature. For example, the concept of heat index can be useful for policy and for explaining results to the public to estimate temperature effects for both temperature and heat index (e.g., Anderson and Bell 2009; Medina-Ramón et al. 2006). Conversely, when investigating the effects of cold weather, there are neither practical nor conceptual reasons to measure exposure with heat index rather than (or in addition to) air temperature: Heat index based only on air temperature and moisture should be very similar or identical to air temperature during the winter months (Figure 3).

Another consideration for environmental health research is whether, physiologically, the heat index is relevant for the population of interest. In developing apparent temperature, Steadman used physiological data (e.g., sweating rates at different temperatures, metabolic energy production rates) from healthy, college-age students (Fanger 1970). He also assumed certain values, such as body dimension [Steadman (1979a) based his calculations on a “model human”: 5’7” (1.7 m) tall and 148 lb (67 kg)]. Heat index therefore may not capture the experience of certain subpopulations. For example, children have a much smaller surface area from which to transfer heat and generally have a higher metabolic rate per surface area (Wenger 2003). Adults’ metabolic rates change with pregnancy or physical exertion (Wenger 2003). Sweating rate can increase substantially with acclimatization or, conversely, be low in the elderly and those with congestive heart failure (Burch and Ansari 1968; Wenger 2003). Sweating can also be affected by prescription drugs, including some antihistimines, sleep aids, and anticholinergics (Wenger 2003). Finally, Steadman’s “model human” dimensions no longer represent the average American—currently the average U.S. male is 5’9’’ and 195 lb, and the average U.S. woman is 5’4’’ and 165 lb (Centers for Disease Control and Prevention 2011).

As a final consideration, like any metric measuring outdoor conditions, heat index does not describe the actual conditions experienced by an entire population, because people spend different amounts of time outdoors, have different levels of activity, and the like. It will therefore have the same uncertainties related to measuring community exposure as any other outdoor weather metric.

This analysis was limited to heat index algorithms used to approximate Steadman’s apparent temperature. Other indices of heat exposure are sometimes used in environmental health research, including the humidex (Environment Canada 2013) and the UTCI (2012). It is possible that some of these other indices of heat exposure may also be calculated using algorithms that vary across studies, so there may be similar concerns for these metrics as the concerns explored in this review for the heat index. Future research could explore whether multiple algorithms are also used to calculate these indices.

## Conclusions

Comparisons among environmental health studies are complicated by differences among model choice, controls for confounding, and exposure metric. Because heat index can be calculated using > 20 different algorithms, the choice of heat index algorithm could further complicate comparisons between studies. However, we found that most heat index algorithms produce values similar to Steadman’s apparent temperature, and that values calculated from one algorithm are generally well correlated with those from other algorithms. Because of this strong agreement between heat index algorithms, most environmental health studies should produce comparable results regardless of the heat index algorithm chosen.

There are, however, exceptions. A few algorithms varied from Steadman’s apparent temperature and from other algorithms when used for cool weather or very low relative humidity. A few others were inconsistent in all weather conditions. Careful selection of a heat index algorithm can help avoid these inconsistencies, and we provide open-source software to implement an algorithm that performs well for all weather conditions.

## Supplemental Material

(119 KB) PDF
