# Supplementary material for: Methods to Calculate the Heat Index as an Exposure Metric in Environmental Health Research
Source: Environ Health Perspect. 2013 Aug 9;121(10):1111–9. doi: 10.1289/ehp.1206273 (PMC3801457; doi:10.1289/ehp.1206273)
Supplement: (119 KB) PDF [file ehp.1206273.s001.pdf]

Environmental Health Perspectives doi:10.1289/ehp.1206273

Supplemental Material

Methods to Calculate the Heat Index as an Exposure Metric in Environmental Health Research

G. Brooke Anderson, Michelle L. Bell, and Roger D. Peng

Table S1. Identifications of weather stations providing weather data for each of the 50 US state capitals for 2011.

State	Capital city	Weather station
Alabama	Montgomery	KMXF
Alaska	Juneau	PAJN
Arizona	Phoenix	KPHX
Arkansas	Little Rock	KJKL
California	Sacramento	KSAC
Colorado	Denver	KAPA
Connecticut	Hartford	KHFD
Delaware	Dover	KDOV
Florida	Tallahassee	KTLH
Georgia	Atlanta	KPDK
Hawaii	Honolulu	PHNL
Idaho	Boise	KBOI
Illinois	Springfield	KSPI
Indiana	Indianapolis	KIND
Iowa	Des Moines	KDSM
Kansas	Topeka	KTOP
Kentucky	Frankfort	KFFT
Louisiana	Baton Rouge	KBTR
Maine	Augusta	KAUG
Maryland	Annapolis	KNAK
Massachusetts	Boston	KBOS
Michigan	Lansing	KLAN
Minnesota	Saint Paul	KSTP
Mississippi	Jackson	KHKS
Missouri	Jefferson City	KJEF
Montana	Helena	KHLN
Nebraska	Lincoln	KLNK
Nevada	Carson City	KRNO
New Hampshire	Concord	KCON
New Jersey	Trenton	KTTN
New Mexico	Santa Fe	KSAF
New York	Albany	KALB
North Carolina	Raleigh	KRDU
North Dakota	Bismarck	KBIS
Ohio	Columbus	KOSU
Oklahoma	Oklahoma City	KPWA
Oregon	Salem	KSLE
Pennsylvania	Harrisburg	KMDT
Rhode Island	Providence	KPVD
South Carolina	Columbia	KCUB
South Dakota	Pierre	KPIR
Tennessee	Nashville	KBNA

State	Capital city	Weather station
Texas	Austin	KATT
Utah	Salt Lake City	KSLC
Vermont	Montpelier	KMPV
Virginia	Richmond	KRIC
Washington	Olympia	KOLM
West Virginia	Charleston	KCRW
Wisconsin	Madison	KC29
Wyoming	Cheyenne	KCYS
